# PCV13 Vaccination of Adults against Pneumococcal Disease: What We Have Learned from the Community-Acquired Pneumonia Immunization Trial in Adults (CAPiTA)

**DOI:** 10.3390/microorganisms10010127

**Published:** 2022-01-08

**Authors:** Christian Theilacker, Mark A. Fletcher, Luis Jodar, Bradford D. Gessner

**Affiliations:** 1 Pfizer Vaccines, 500 Arcola Rd., Collegeville, PA 19426, USA; Luis.Jodar@pfizer.com (L.J.); Bradford.gessner@pfizer.com (B.D.G.); 2 Pfizer Emerging Markets, 23-25 Avenue du Docteur Lannelongue, 75014 Paris, France; Mark.A.Fletcher@pfizer.com

**Keywords:** Community-Acquired Pneumonia immunization Trial in Adults (CAPiTA), pneumococcal disease, 13-valent pneumococcal conjugate (PCV13) vaccine, community-acquired pneumonia, invasive pneumococcal disease

## Abstract

The Community-Acquired Pneumonia immunization Trial in Adults (CAPiTA) evaluated older adult pneumococcal vaccination and was one of the largest vaccine clinical trials ever conducted. Among older adults aged ≥65 years, the trial established 13-valent pneumococcal conjugate vaccine (PCV13) efficacy in preventing first episodes of bacteremic and nonbacteremic pneumococcal vaccine serotype (VT) community acquired pneumonia (CAP), and of vaccine serotype invasive pneumococcal disease (VT-IPD). Since the publication of the original trial results, 15 additional publications have extended the analyses. In this review, we summarize and integrate the full body of evidence generated by these studies, contextualize the results in light of their public health relevance, and discuss their implications for the assessment of current and future adult pneumococcal vaccination. This accumulating evidence has helped to better understand PCV13 efficacy, serotype-specific efficacy, efficacy in subgroups, the interpretation of immunogenicity data, and the public health value of adult PCV vaccination.

## 1. Introduction

*Streptococcus pneumoniae* is an important cause of morbidity and mortality globally, in both children and adults, and accounts each year for an estimated 660,000 lower respiratory tract infection (LRTI)-related deaths and 9600 deaths due to meningitis in adults aged ≥50 years of age globally [1,2]. *S. pneumoniae* has been a leading cause of invasive disease (such as bacteremia or sepsis, bacteremic pneumonia, meningitis, and septic arthritis) and of noninvasive mucosal disease (such as nonbacteremic pneumonia, sinusitis, and acute otitis media), with a large global burden in both high- and low-resource countries [3,4]. *S. pneumoniae* has 100 distinct serotypes that differ in their propensity to cause disease overall, as well as their association with case fatality, and antimicrobial resistance [5,6]. Adult pneumococcal disease incidence increases substantially after 50 years of age, as does associated mortality. In addition to age as a risk factor, smoking and certain medical conditions, including immunocompromising conditions, also increase the risk for pneumococcal infection and are associated with potentially increased morbidity and mortality [7,8,9].

Until recently, two vaccines have been available for protection against pneumococcal infections in adults: a 23-valent pneumococcal polysaccharide vaccine (PPSV23) and a 13-valent pneumococcal conjugate vaccine (PCV13) [10]. PPSV23 was licensed in 1983 based on trials demonstrating the efficacy of precursor formulations of lower valency and higher antigen content against bacteremic community-acquired pneumonia (CAP) in South African gold miners and Papua New Guinean highlanders [11]. Since then, post-licensure studies evaluating PPSV23 effectiveness against invasive pneumococcal disease (IPD) have confirmed its ability to protect against IPD, while evidence on the prevention of nonbacteremic pneumococcal pneumonia in older adults has been inconclusive [10,12]. 

PCV13 is a 13-valent conjugate vaccine that contains capsular polysaccharides from serotypes 1, 3, 4, 5, 6A, 6B, 7F, 9V, 14, 18C, 19A, 19F, and 23F individually conjugated to genetically detoxified diphtheria toxin, CRM197, by reductive amination. Licensing pathways of PCV13 for the adult indication differed between the US Food and Drug Administration (FDA) and the EU European Medicines Agency (EMA). The FDA approved PCV13 in 2011 for prevention of both pneumonia and IPD caused by PCV13 serotypes among adults aged 50 years and older [13]. Approval was given under the Accelerated Approval pathway, which allows the agency to approve products for serious or life-threatening diseases based on early evidence of a product’s effect that is reasonably likely to predict clinical benefit. Approval of PCV13 for adults was based on immunogenicity studies that compared immune responses between PCV13 and PPSV23. It was noted, however, that no consensus existed regarding PPSV23 protection against nonbacteremic pneumococcal pneumonia, and Pfizer was required to conduct a clinical endpoint efficacy trial to verify the clinical benefit of PCV13 vaccination [13]. To meet this post-approval commitment, CAPiTA (Community-Acquired Pneumonia immunization Trial in Adults, referred to as “the trial” from now on) was undertaken to establish PCV13 efficacy against vaccine serotype (VT) pneumococcal CAP, as well as VT IPD, in adults ≥65 years of age [14]. In the EU, PCV13 was approved in 2011 only for the prevention of IPD among adults aged ≥50 years [15]. The EMA also concluded that PPSV23 efficacy against non-invasive CAP had not been demonstrated consistently and therefore did not grant licensure for a pneumonia indication based on establishing an immunobridge between PCV13 and PPSV23. Once the positive results of the trial became available in 2014/2015, the EMA approved the extension of the PCV13 label to include an adult indication for the prevention of pneumococcal CAP caused by vaccine serotypes [16]. 

Since then, PCVs with broader serotype formulations have entered clinical development for an adult indication, with a 20-valent PCV (PCV20) having completed phase 3 clinical trials [17,18,19,20]. PCV20 includes serotypes 8, 10A, 11A, 12F, 15B, 22F, and 33F, in addition to the PCV13 serotypes [19]. In this setting, the FDA has granted licensure to PCV20 for adults ≥18 years of age for the prevention of pneumonia and invasive disease caused by the 20 vaccine serotypes. The IPD indication was based on immunologic comparability to PCV13 for the 13 shared serotypes and to PPSV23 for the 7 additional serotypes. For the pneumonia indication, PCV20 effectiveness for the 13 shared serotypes was supported by comparable OPA antibody responses to PCV13 and by nearly identical manufacturing processes for PCV20 and PCV13 vaccines. For the 7 new vaccine serotypes, Accelerated Approval was granted for the pneumonia indication, based on an immunological surrogate endpoint (OPA titer) considered reasonably likely to predict prevention of pneumococcal pneumonia caused by these serotypes. To confirm this predicted prevention, Pfizer committed to a post-approval, real-world, observational PCV20 effectiveness study for the 7 new vaccine serotypes [21]. 

The current review brings together the results for the primary, secondary and exploratory objectives of the trial with data from 15 additional publications that have enlarged the scope of the initial publication by describing the methods used in the trial, by presenting exploratory endpoint results, and by presenting results for various post-hoc analyses (Supplementary Table S1). This integrated analysis in turn should contribute to our understanding of the PCV20 licensure pathway (since PCV20 licensure is based in part on bridging to PCV13 data from the trial), to understand more completely the full public health value of PCV13 when used in an older adult population in the context of a robust pediatric PCV program, and to interpret results from observational studies of PCV13. 

## 2. Materials and Methods

### 2.1. Trial Design and Participants

In the trial, the efficacy and safety of PCV13 was assessed in 84,496 immunocompetent, community-dwelling individuals of age 65 years and above living in The Netherlands [14]. The trial was conducted between 2008 and 2014 and used a parallel-group, randomized, double-blind, placebo-controlled design (for details see Table S1). With the introduction of PCV7 infant vaccination in The Netherlands in 2007 and its replacement by PCV10 in 2011, the trial was conducted in a setting of ongoing indirect effects from pediatric PCV vaccination [22]. Trivalent influenza vaccine was coadministered by general practitioners with the study vaccine in 30.4% of study participants [14], a level of influenza vaccine uptake that is comparable with many other European countries [23].

Among study participants, 69% were aged 65 to 74 years, 28% 75 to 84 years, and 4% ≥85 years, an age distribution that was slightly younger than that of the general population in The Netherlands at the time [24]. Based on self-reported comorbidities, 49% of the total study population had at least one medical condition considered to predispose to pneumococcal disease, with heart disease (25.3%) being the most prevalent condition, followed by diabetes (12.5%), smoking (12.3%), lung disease (10.2%), asthma (4.9%) and liver disease (0.5%) [25]. 

The trial was event-driven, aiming to capture at least 130 first episodes of VT CAP hospitalizations, and surveillance for suspected cases of CAP and IPD was performed in 101 referral hospital or diagnostic center sites located in the study area. The modified intention-to-treat (mITT) efficacy population included participants who had an episode of CAP or IPD with the onset of symptoms ≥14 days after vaccination. The per-protocol population (PP) excluded mITT participants with a protocol-violation or participants presenting with immunocompromising conditions, post-obstructive pneumonia due to cancer, *Pneumocystis jirovecii* pneumonia or active tuberculosis. 

### 2.2. Objectives and Procedures

The main goal of the trial was to either confirm or inform licensure for the prevention of CAP in adults by providing an accurate estimate of direct PCV13 protection against VT CAP, nonbacteremic/noninvasive (NB/NI) VT CAP and VT IPD as target etiologies. A major challenge for the trial design was therefore to find a diagnostic test for NB/NI VT CAP that maximized specificity to provide an unbiased estimate of efficacy [26], while maintaining reasonable sensitivity. This excluded sputum culture testing for *S. pneumoniae* from the lower respiratory tract (insufficient specificity) and the pneumococcal urinary C-wall polysaccharide antigen testing (BinaxNOW S. pneumoniae^®^, lack of discrimination of individual serotypes). Consequently, a novel multiplex immunoassay called serotype-specific urinary antigen detection test (UAD) had to be developed to identify NB/NI VT CAP. The UAD is a multiplex assay based on Luminex technology that captures pneumococcal serotypes antigens excreted in the urine for each of the 13 serotypes contained in PCV13 [27]. Positivity cut-off limits for individual serotypes are calibrated using control urines from healthy adults from the same population. Positivity cut-offs are optimized for specificity and always set to achieve at least 97% specificity per serotype [27,28]. In a pilot study for the trial, the UAD assay demonstrated 100% specificity and 97% sensitivity compared to a gold standard of bacteremic CAP [28]. 

## 3. Results and Discussion

### 3.1. The Trial Demonstrated PCV13 Efficacy for Prevention of First Episodes of VT CAP, Nonbacteremic/Noninvasive VT CAP and VT IPD

The trial enrolled subjects between September 2008 and January 2010, and follow-up for endpoint detection continued until August 2013, resulting in a mean follow up of 3.9 years (min 3.6, max 4.9 years). The primary objective was to assess PCV13 efficacy in preventing first episodes of confirmed VT pneumococcal CAP in adults aged ≥65 years. Secondary objectives included demonstrating PCV13 efficacy in preventing first episodes of confirmed NB/NI VT pneumococcal CAP and first episodes of VT-IPD. 

In the per-protocol analysis of first episodes of VT outcomes, 49 participants in the PCV13 group and 90 in the placebo group developed CAP for a vaccine efficacy (VE) of 45.6% (95% confidence interval [CI] 21.8 to 62.5) against VT CAP. NB/NI VT CAP was reported in 33 PCV13 group participants and 60 placebo group participants (VE, 45.0%; 95% CI 14.2 to 65.3) while VT IPD was reported in 7 PCV13 group participants and 28 placebo group participants (VE, 75.0%; 95% CI 41.4 to 90.8). For VT CAP, NB/NI VT CAP, and VT IPD, respectively, PCV13 demonstrated similar efficacy in the mITT population for first VT episodes (VE, 37.7%, 41.1% and 75.8%) and all VT episodes (VE 37.5%, 39.2% and 75.8%) [14,29,30]. 

The post-hoc analysis by Patterson et al. performed Kaplan–Meier time-to-event analyses of the primary and secondary endpoints and derived VE behavior over time [31]. This analysis confirmed that cases of VT CAP, NB/NI-VT CAP, and VT IPD over the full postvaccination study period were greater in subjects who received the placebo compared with subjects who received PCV13 and showed a significant difference in disease-free survival between PCV13 and placebo recipients for these outcomes [31]. Hence, VE did not decline during the mean follow-up time of approximately 4 years, and when taking into account endpoint episodes occurring during the time of vaccination (2008 to 2010), VE did not decline over 4.9 years [14,31]. 

### 3.2. The Trial Results Put into Context—What Have We Learned over Time?

Several post-hoc analyses described VE in special populations or by individual serotype. Additional analyses addressed the impact of the PCV13 vaccination on outcomes relevant to public health by applying an analytical framework to the trial data that is more appropriate for vaccine impact assessments. A substudy measured PCV13 immune responses in a subgroup of trial participants and assessed their relation to clinical protection. A summary of the 15 main publications related to the trial is given in supplementary Table S1.

### 3.3. PCV13 Efficacy with Chronic Medical Conditions 

Two post-hoc analyses of the trial data examined PCV13 VE among persons with comorbidities using slightly different methodologies [25,32]. When using self-reported comorbidity status at study entry, both publications reported identical point estimates against VT CAP, including 40.3% (95% CI 11.4% to 60.2%) among study subjects with at-risk conditions versus 66.7% (95% CI 11.8% to 89.3%) among subjects without risk conditions. The analysis of Huijts et al. assessed VE based on medically-documented comorbidities and reported similar PCV13 VE for participants with or without underlying comorbidities (VE 45.3%; 95% CI 19.9 to 62.6 and 46.7%; 95% CI −25.8 to 77.4, respectively). This analysis also assessed effect modification of PCV13 efficacy by selected comorbidities and concluded that PCV13 efficacy was modified by diabetes mellitus leading to higher VE (*p* value for interaction 0.002) and by respiratory disease leading to lower VE (*p* value for interaction 0.054). As in any post-hoc analysis, however, the randomization process may not have controlled for confounders on a substratum level, and multiple comparisons without multiplicity adjustments may have led to spurious results. Based on a recent literature review, there is little evidence to support the role of diabetes as a positive effect modifier for vaccines targeting influenza, pneumococcus, and varicella zoster in adults [33]. No studies examining the interaction of chronic respiratory disease with the effectiveness of vaccines for the prevention of respiratory infection could be identified by the authors of this review. 

### 3.4. The Trial Demonstrated PCV13 Protection up to 84 Years of Age but Was Underpowered to Measure VE in Older Persons

One post-hoc analysis of the trial examined the interaction effect of age at enrolment, with the first episode of disease, either VT-CAP or VT-IPD [34]. PCV13 efficacy estimates were 49.3% (95% CI 26.2 to 67.1) for adults aged 65 to 74 years and 40.5% (95% CI 3.3 to 65.9) for adults aged 75 to 84 years. VE was not demonstrated among persons aged ≥85 years, but with only 12 total VT-CAP episodes in this group study power was limited. 

### 3.5. Totality of Data Are Consistent with PCV13 Efficacy against Serotype 3 Similar to the Overall PCV13 Efficacy

PCV13 has been introduced into pediatric national immunization programs in many countries globally with high uptake and in the USA and other countries also with programs targeting older adults [35,36]. Nevertheless, serotype 3 continues to be among the most prevalent serotypes causing IPD in adults [37,38,39,40]. This raises the question of whether PCV13 has limited ability to reduce serotype 3 carriage (and thus provide indirect protection), limited ability to provide direct protection against this serotype in vaccinated persons, or both. Although the trial was not powered to measure VE for each of the individual PCV13 serotypes, the number of cases due to serotypes 1, 3, 6A, 7F, and 19A were sufficient to allow for calculation of VE estimates. Serotype 3 was the second most prevalent serotype in the placebo group, accounting for 20 cases in the mITT population. PCV13 effectiveness for serotype 3 was 60.0% (95% CI 5.2% to 84.8%) against first episodes of radiologically confirmed CAP and 61.5% (95% CI 17.6% to 83.4%) against all-episodes of clinical CAP [41]. A reanalysis of the trial data that used hierarchical modeling for estimation of serotype-specific efficacy of PCV yielded almost identical results [42]. Data from the trial was included in a pooled analysis, which also incorporated data from test-negative design VE studies from Louisville (Kentucky, USA) and Argentina, with a reported VE against serotype 3 CAP of 52.5% (95%CI: 6.2–75.9) [43]. These data indicate PCV13 provided protection against serotype 3 CAP among directly vaccinated older adults. 

### 3.6. Rate Reductions Reported for the Primary and Secondary Endpoints Underestimated the Full PCV13 Impact

Initial evaluations of PCV13 impact used vaccine serotype-specific endpoints designed to support the regulatory objective of demonstrating vaccine efficacy. However, this objective sacrificed the public health goal of accurately estimating overall impact obtained by calculating rate reductions from more sensitive all-cause outcomes [44,45]. A recent systematic literature review that compared rate reductions by outcome definition for PCV vaccination of children or adults documented that studies using microbiologically-confirmed outcomes, under-ascertained vaccine impact by 1.5- to 4-fold, and concluded that clinically-defined outcomes provide more accurate estimates of PCV13 public health impact both in children and adults [45]. This evaluation used a measure of public health called vaccine-preventable disease incidence (VPDI), which is calculated by subtracting the incidence rate of the outcome in the vaccinated group from the incidence rate in the placebo group; this is mathematically equivalent to the VE multiplied by the placebo group incidence [44]. A related measure is the number needed to vaccinate (NNV), which is the reciprocal of VPDI divided by duration of vaccine protection. 

A re-evaluation of PCV13 impact in the trial population was conducted following an analytic framework for public health impact evaluations [30]. Specifically, the evaluation measured VPDI and NNV considering all events in the mITT population and using protocol-defined clinical CAP as the outcome [44,46,47]. This evaluation was complemented by another post-hoc analysis that reported PCV13 efficacy for clinical CAP and lower respiratory tract infections (LRTI) in participants that were treated in primary care [46,48]. Both analyses documented VPDIs for clinically-defined disease that were much larger than those reported for etiologically-confirmed outcomes. Compared to hospitalized VT CAP, VPDIs for hospitalized, radiologically-confirmed CAP and clinical CAP, independent of specific radiologic findings, were 1.5-fold and 2.9-fold higher, respectively (Table 1). For overall clinical CAP and LRTI, regardless of the treatment setting, VPDI was 10-fold and 22.8-fold higher compared to the VPDI for hospitalized VT CAP (Table 1). 

Corresponding NNVs were also highly favorable, with NNVs of 80 and 39 to prevent one episode of clinical CAP and LRTI, respectively (Table 1). As noted above, this impact was in addition to indirect benefits through use of PCV7 followed by PCV10 in the pediatric national immunization program. These NNVs are not dissimilar to other vaccines recommended for adults, such as the herpes zoster subunit vaccine for which NNVs of 11 to 17 for the prevention of a herpes zoster case and NNVs of 70 to 187 for the prevention of one case of post-herpetic neuralgia have been estimated [49].

Assuming a duration of protection against CAP of at least 5 years [31], a PCV13 uptake of 100% among all adults age ≥ 65 years living in the Netherlands would have prevented 32,113 cases of CAP and 73,034 cases of LRTI in the primary and secondary care sector between 2008 and 2013 (Table 1).

The substantially higher VPDIs seen for clinically-defined outcomes versus those seen for hospitalized VT-CAP likely occurred for several reasons. First, missed pneumonia episodes led to an underestimation of the NNV based on the primary trial results [50], as illustrated by a post-hoc analysis that evaluated the completeness of outcome capture. This analysis searched GP records during the trial surveillance period for hospital referrals based on a diagnosis of suspected pneumonia, and then assessed whether these episodes were captured as study endpoints in the trial. The analysis demonstrated that 63% of the suspected pneumonia episodes among trial participants were identified, suggestive of a 37% under-ascertainment of the absolute number of endpoint episodes prevented. Episodes were missed because they were not identified by participating hospital sites or because participants were treated outside the screened routes. While important, this would explain only a small proportion of the under-ascertainment in rate reductions. Second, a requirement for etiologic confirmation will underestimate the preventable disease burden to some degree due to failure to obtain diagnostic specimens and limited test sensitivity. For example, the UAD used in the trial to identify serotype specific CAP, has an unknown sensitivity for NB/NI VT CAP because of the lack of an accepted gold standard [28]. Finally, etiologically confirmed outcomes may underestimate rate reductions if the pneumococcus is part of the causal chain for CAP but no longer present in sufficient quantity to be detected by the diagnostic test at the time of clinical presentation [51]. 

### 3.7. Broader Value of PCV13 Vaccination—Impact on Mortality Outcomes, Antibiotic Consumption and Healthcare Utilization

In the publication by Bonten et al. [14] that presented the results for primary and secondary endpoints of the trial, PCV13 vaccination did not prevent deaths from VT-CAP/IPD or from all-cause CAP. However, in the trial, death associated with VT CAP was rare, while the specificity of all-cause CAP-associated death was low, leading to underpowered analyses for both outcomes. While not included in the presentation of primary study results, the trial also captured the protocol-prespecified outcome of infection-associated death, an outcome that was more specific than all-cause CAP and that was reasonably common among study participants. In a post-hoc analysis, the PCV13 VE for infection-associated death was 14.7% (95% CI −5.5% to 31.1%) for the overall study population and 21.4% (95% CI −0.4% to 38.4%) among study participants with underlying at-risk conditions, consistent with a reduction in infection-associated deaths, presumably the only category of deaths PCV13 can prevent [30]. 

Similar to observations of PCV13 impact in pediatric populations [52,53], the trial data also indicated that PCV13 vaccination may reduce antibiotic prescriptions for LRTIs in older adults treated in primary care (Table 1). Although the relative reduction in antibiotic use for LRTI was modest, the absolute rate reduction of 480 LRTI-related antibiotic prescriptions per 100,000 patient years of observation was substantial (Table 1). The previously described public health analysis also demonstrated that for the outcome of hospitalized clinical CAP, PCV13 vaccination resulted in a reduction of 909 days spent in hospital and 145 days spent in the ICU per 100,000 patient years of follow up, with corresponding NNVs to prevent one hospital or ICU day of 22 and 145, respectively [30,54]. The finding that PCV13 vaccination reduces the utilization of healthcare resources needed for the treatment of severe respiratory infection could be of particular interest in the context of strained healthcare system resources limited during respiratory infection epidemics or pandemics.

### 3.8. Within the Trial Population, Opsonophagocytic Killing Assay (OPA) Immune Responses Did Not Predict Duration or Extent of Protection 

Correlates of protection (CoPs) are important in clinical vaccine development for several reasons, including the validation of vaccines for which efficacy trials are not ethical, as when a prior-generation vaccine is already licensed and the standard of care [55]. In an attempt to establish a CoP for PCV13 protection against VT CAP in adults, an immunogenicity substudy was nested into the trial, with serotype specific OPA and immunoglobulin G (IgG) responses measured before vaccination, and at 6, 12, and 24 months after PCV13 vaccination, in 1006 participants per study arm [56]. Unfortunately, no CoP could be inferred because too few data points were available to validate cut-off levels. 

Nevertheless, the trial did allow for comparisons of PCV13 immunogenicity with clinical protection on a population-level. A single dose of PCV13 induced robust OPA geometric mean titers (GMTs) and serotype-specific IgG concentrations 30-days post-vaccination. Although immune responses remained numerically higher compared to baseline levels during the two-year observation period, a substantial waning of OPA titers was observed, with OPA GMTs only minimally above baseline at 24 months post vaccination for most PCV13 serotypes. Both the magnitude of OPA GMT responses one-month post-vaccination, as well as the persistence of functional antibody responses over time, showed no correlation with clinical protection on a population level either overall or by individual serotypes. Specifically, sustained protection was demonstrated against VT-CAP for serotypes 3, 19A and 7F for at least 4.9 years despite near baseline OPA titers by study year 2 [31,41,56]. The lack of correlation was most pronounced for serotype 3, for which the reverse cumulative distribution curves at 24 months post-vaccination demonstrated OPA titers that were below the lower limit of quantification in 62% of the subjects despite robust CAP protection over the full study period [41]. These results suggest that neither initial nor long-term OPA responses predicted long-term protection against clinical disease such as CAP and strongly suggest that unmeasured immune responses, such as mucosal or humoral and cellular immunity, may be critical for this outcome. 

## 4. Conclusions

The Community-Acquired Pneumonia immunization Trial in Adults was one of the largest adult vaccination randomized control trials (RCTs) ever conducted, and it remains the only RCT of a PCV to provide VE estimates for adults aged ≥65 years, the primary adult target of pneumococcal vaccines. By determining VT-CAP with the validated and highly specific UAD assay, it is the only pneumococcal vaccine RCT that has demonstrated vaccine efficacy against vaccine serotype pneumococcal NB/NI CAP [14]. A total of 15 publications further complement the results reported for the primary objectives of the trial (Supplemental Table S1). These analyses showed similar VE in study participants with underlying chronic medical conditions compared to individuals without these conditions [25,32]; they also demonstrated that protection against VT pneumococcal disease was maintained with increasing age, as demonstrated in participants up to 84 years of age [34]. Furthermore, although PCV13 immunogenicity in adults ≥80 years of age was somewhat lower compared to younger trial participants, PCV13 in this age group generally induced robust OPA responses that were supportive of clinical effectiveness [56]. The efficacy reported for serotype 3 CAP using three different analytical approaches was consistent with overall PCV13 efficacy against VT CAP [41,42,43].

The trial findings for PCV13 VE against serotype specific CAP were subsequently confirmed by a real-world effectiveness study using a test-negative design in US adults aged ≥65 years [57]. With more than one third of study participants aged ≥80 years and a median age of 76 years, this study provided a representative sample of the age distribution in the US general population of older adults, which also includes individuals underrepresented in the trial study population, such as immunocompromised persons or persons over age 80 years [57]. The PCV13 VE against VT-CAP in the Louisville Pneumonia study was 72.8% (95% CI 12.8 to 91.5), demonstrating that the Netherlands trial VE estimates are externally valid and may be extrapolated to the general population of older adults.

Initially, public health assessments from the trial data were based on results reported for the primary and secondary endpoints, leading to questions about whether vaccine impact would be sufficient to warrant universal recommendation [58,59]. However, these assessments used etiologically-confirmed outcomes, which, due to their lower sensitivity (as compared to higher specificity), were not suitable to quantify the full public health impact in the trial population. Two reanalyses of the trial data from a public health lens assessed PCV13 impact on clinically-defined outcomes and demonstrated a much larger impact of PCV13 vaccination, especially if outcomes that occurred in the primary care setting were also considered [30,46]. Studies other than CAPiTA have also documented that in adults, rate reductions against all-cause pneumonia may be multiples higher than those for etiologically-confirmed disease [46]. 

Due to the randomized vaccine allocation and placebo-controlled design, the rate reductions measured in the trial reflect an unbiased estimate of the true vaccine impact in The Netherlands during the time the study was conducted. In contrast, real-world vaccine impact evaluations of PCV13 adult vaccination on IPD or CAP are more challenging to interpret, as vaccine impact measured by surveillance systems is a combination of disease reduction in directly vaccinated individuals and disease reduction among unvaccinated persons due to less carriage among and transmission from vaccinated persons [60]. Furthermore, little is static in real-world populations. For example, changes in healthcare seeking behavior or disease surveillance [61], variability of respiratory virus co-circulation [62], spread of pneumococcal clades with better adaptation to survive the nasopharyngeal niche as reported for serotype 3 [63], or changes in adult or pediatric PCV uptake may complicate extrapolation of the trial results to other populations, direct comparison of the trial results to other studies, or translating well-documented VE among directly vaccinated persons to population level reductions among older adults.

The trial has also had important implications for the pathway to licensure for next-generation pneumococcal vaccines for adults. With the establishment of PCV13 efficacy against VT pneumococcal disease, trials of future pneumococcal vaccines may not be able to use placebo-controlled RCTs with clinical endpoints, both due to excessive sample size as well as ethical considerations in settings where PCV13 is licensed and recommended. Instead, effectiveness of future PCVs against pneumococcal CAP, as the most important clinical presentation of pneumococcal disease in adults, needs to be inferred from CAPiTA on the basis of immunologic and physicochemical comparability with PCV13, a licensed vaccine with proven efficacy against this outcome. 

## Figures and Tables

**Table 1:** Incidence rates and vaccine-preventable disease incidence rate (VPDI) per 100,000 person-years of observation and numbers needed to vaccinate (NNV) based on 5 years of 13-valent pneumococcal conjugate vaccine (PCV13) duration of protection against pneumonia.

Endpoint	IR, Unvaccinated *	IR, Vaccinated *	VE, % (95% CI)	VPDI *	NNV	Extrapolation to The Netherlands, 2008 to 2013	
						Total Number of Outcomes over 5 years ^‡^	Total Number of Averted Outcomes ^§^
Secondary care [30]							
VT-IPD	20	5	75.8 (47.6, 90.3)	15	1342	2537	1923
VT-CAP	67	42	37.5 (14.3, 54.5)	25	798	8602	3226
Radiologically-confirmed CAP	559	522	6.7 (−4.1, 16.3)	37	535	72,009	4825
Clinical CAP	891	819	8.1 (−0.6, 16.1)	72	277	114,761	9296
Primary care [46,48]							
Clinical CAP	2020	1910	5.5 (−2.6, 13.0)	110	182	260,118	14,306
LRTI	11,550	11,120	3.8 (−1.1, 8.4)	430	47	1,487,306	56,518
LRTI-related antibiotic prescriptions	11,270	10,790	4.2 (−1.0, 9.1)	480 ^†^	42 ^†^	1,451,250	60,952
Primary and secondary care combined [46]							
Clinical CAP	3370	3120	7.4 (0.0, 14.4)	250	80	433,958	32,113
LRTI	12,890	12,320	4.4 (−0.3, 9.0)	570	39	1,659,859	73,034

NOTES: IR, incidence rate, 95% VE: vaccine efficacy; CI: 95% confidence interval; VPDI: vaccine-preventable disease incidence; NNV: numbers needed to vaccinate (to prevent one outcome episode over a 5-year period for adults); VT: Vaccine serotype; IPD: invasive pneumococcal disease; CAP: community-acquired pneumonia; LRTI: lower respiratory tract infection; * Incidence rates and VPDI are expressed per 100,000 person years of observation. ^†^ calculated by authors based on VE point estimates and 95% confidence intervals reported in the original publication. VPDI was calculated as VPDI = placebo IR – PCV13 IR. 95% CI for VPDI was based on risk difference with binomial distribution, assuming the person-years as total patients all followed for 1 year and calculated using an online calculator (http://vassarstats.net/prop2_ind.html, accessed on 2 July 2021). NNV calculated as NNV = 1/(VPDI × 5) (assuming 5 years of constant protection). ^‡^ Calculated from incidence rate in unvaccinated × cohort size × 5 ÷ 100,000. The cohort size for adults ≥65 years of 2,575,421 was taken from the World population prospects for the Netherlands in 2010 (United Nations Department of Economic and Social Affairs Population Division, World Population Prospects, Available at https://population.un.org/wpp/. accessed 5 November 2021). ^§^ Calculated as total number of outcomes over 5 years × VE.

## Data Availability

Not applicable.

## Supplementary Materials

The following are available online at https://www.mdpi.com/2076-2607/10/1/127/s1, Table S1: Summary of CAPiTA (The Community-Acquired Pneumonia immunization Trial) related publications and analyses.
