# Correction: Theilacker et al. PCV13 Vaccination of Adults against Pneumococcal Disease: What We Have Learned from the Community-Acquired Pneumonia Immunization Trial in Adults (CAPiTA). *Microorganisms* 2022, *10*, 127

**DOI:** 10.3390/microorganisms10051018

**Published:** 2022-05-12

**Authors:** Christian Theilacker, Mark A. Fletcher, Luis Jodar, Bradford D. Gessner

**Affiliations:** 1 Pfizer Vaccines, 500 Arcola Rd., Collegeville, PA 19426, USA; luis.jodar@pfizer.com (L.J.); bradford.gessner@pfizer.com (B.D.G.); 2 Pfizer Emerging Markets, 23-25 Avenue du Docteur Lannelongue, 75014 Paris, France; mark.a.fletcher@pfizer.com

The authors wish to make the following corrections to this paper [1]:

After the publication of the manuscript, the authors recognized an error in the second paragraph of Section 3.1. The Trial Demonstrated PCV13 Efficacy for Prevention of First Episodes of VT CAP, Nonbacteremic/Noninvasive VT CAP and VT IPD. In the published version, it was stated that “For VT CAP, NB/NI VT CAP, and VT IPD, respectively, PCV13 demonstrated similar efficacy in the mITT population for first VT episodes (VE, 37.7%, 41.1% and 75.8%) and all VT episodes (VE 22.2%, 39.2% and 75.8%) [29].”

To the correct version, as follows:

“For VT CAP, NB/NI VT CAP, and VT IPD, respectively, PCV13 demonstrated similar efficacy in the mITT population for first VT episodes (VE, 37.7%, 41.1% and 75.8%) and all VT episodes (VE 37.5%, 39.2% and 75.8%) [14,29,30].”

The authors apologize for any inconvenience caused and state that the scientific conclusions are unaffected. The original publication has also been updated.
