# Supplementary material for: PCV13 Vaccination of Adults against Pneumococcal Disease: What We Have Learned from the Community-Acquired Pneumonia Immunization Trial in Adults (CAPiTA)
Source: Microorganisms. 2022 Jan 8;10(1):127. doi: 10.3390/microorganisms10010127 (PMC8778913; doi:10.3390/microorganisms10010127)
Supplement: Supplementary file 1 [file microorganisms-10-00127-s001.zip › microorganisms-1484126-supplementary.pdf]

Supplementary Table S1: Summary of CAPiTA (The Community-Acquired Pneumonia immunization Trial) related publications and analyses.

Study objective(s)	Study details	Outcomes	Results	Conclusion by authors
**Bonten et al. 2015 (1)**				
**Primary objective-**<br>To evaluate the efficacy of PCV13 in the prevention of first episode of confirmed VT-CAP<br><br>**Secondary objectives-**<br>To evaluate the efficacy of PCV13 in the prevention of first episode of confirmed nonbacteraemic and noninvasive VT-CAP and VT-IPD<br><br>**Pre-specified exploratory end points:**<br>All episodes of confirmed VT-CAP, first episode CAP irrespective of serotype, first episode IPD irrespective of serotype, first episode CAP of any cause and death	**Primary CAPiTA study**<br>Parallel-group, randomised, double-blind, PBO-controlled multicentre trial<br>N= 84,496 older adults ≥65 years<br>○ N (PCV13): 42,240<br>○ N (PBO): 42,256<br><br>**Eligibility criteria-**<br>Inclusion:<br>• Male or female adults ≥65 years<br>• Registered with a GP participating in the study;<br>• Ability to fulfil study requirements.<br>Exclusion:<br>• Previous vaccination with any pneumococcal vaccine<br>• Residence in long-term care facility<br>• Contraindication for PCV13<br>• Contraindication for influenza vaccines<br>• Use of investigational products in 30 days prior to study Vaccine administration<br>• History of severe adverse reaction associated with any vaccine component<br>• Immunodeficiency or immune suppression including human immunodeficiency virus infection, leukaemia, lymphoma, Hodgkin disease, multiple myeloma, generalised malignancy, chronic renal failure, receipt of immunosuppressive therapy, or an organ or bone marrow transplant.<br><br>**Duration of follow-up-**<br>3.97 years (mean), subjects were enrolled between September 2008 and January 2010 and follow-up was continued up to August 2013<br><br>**Discontinuations, N (%) -**<br>PCV13: 5,238 (12.4%)<br>PBO: 5324 (12.6%)	• Clinical and radiological criteria, standard laboratory methods and a SS UAD assay were used to identify CAP and IPD<br><br>• mITT population: Participants who had an episode of CAP or IPD with the onset of symptoms at least 14 days after vaccination. The mITT was further categorised according to the participant's immune status at the time of presentation with CAP or IPD.<br>○ immunodeficient or immunosuppressed persons;<br>○ immunocompetent persons<br><br>• PP population: Participants meeting criteria for mITT; eligible for the study; received a vaccination; and had no other major protocol violations. Episodes of CAP and IPD were excluded from the PP analyses if the onset of symptoms occurred after receipt of any nonstudy pneumococcal vaccine or after any of the following diagnoses: immunodeficiency or immunosuppression, bronchial obstruction due to primary lung cancer, another cancer that was metastatic to the lungs, postobstructive pneumonia, AIDS or known or suspected *Pneumocystis jiroveci* pneumonia or active tuberculosis.	• PP analysis of first episodes of infections due to VT strains:<br>○ CAP occurred in 49 persons in the PCV13 and 90 persons in the PBO (VE, 45.6%; 95.2% CI, 21.8 to 62.5)<br>○ Nonbacteraemic and noninvasive CAP occurred in 33 persons in the PCV13 and 60 persons in the PBO (VE, 45.0%; 95.2% CI, 14.2 to 65.3)<br>○ IPD occurred in 7 persons in the PCV13 and 28 persons in the PBO (VE, 75.0%; 95% CI, 41.4 to 90.8)<br><br>• In the mITT analysis, similar efficacy was observed (VE, 37.7%, 41.1%, and 75.8%, respectively), and CAP occurred in 747 persons in the PCV13 group and 787 persons in PBO group (VE, 5.1%; 95% CI, −5.1 to 14.2)<br><br>• Efficacy persisted throughout the trial.<br><br>• No. of SAEs and deaths were similar in the two groups, but there were more local reactions in the PCV13.	• PCV13 was effective in preventing VT pneumococcal, bacteremic, and nonbacteraemic CAP and VT-IPD but not in preventing CAP from any cause

Study objective(s)	Study details	Outcomes	Results	Conclusion by authors
	• Discontinuations were primarily due to deaths (7.1% in each group) and loss to follow-up<br><br>**Loss to follow-up, N (%) -**<br>PCV13: 2027 (4.8%)<br>PBO: 2155 (5.1%)			
**Werkhoven et al. 2015 (2)**				
To analyse the impact of age on the observed VE of PCV13 in the prevention of VT-CAP and VT-IPD in immunocompetent CAPiTA participants	**Post-hoc analysis CAPiTA (whole cohort)**<br>see Bonten 2015<br><br>**Eligibility criteria**<br>see Bonten 2015<br><br>**Duration of follow-up**<br>see Bonten 2015<br><br>**Loss to follow-up, N (%) -** 4571 (5.4%), whole cohort<br><br>**Deaths, N (%)** - 6011 (7.1%), whole cohort	• Proportions of patients with blood cultures and SS UAD tests were compared over age groups and between the vaccine and PBO group.<br><br>• The interaction effect of age at enrolment was assessed using a Cox proportional hazard model, with the first episode of either VT-CAP or VT-IPD as the outcome variable.<br><br>• Subjects that had died were censored at time of death.<br><br>• Because subjects that were lost to follow-up could still be identified as CAP or IPD case if they presented in a participating hospital, loss to follow-up was ignored.	• Age was similarly distributed in the vaccine and PBO group with a median of 71.6 years (IQR 68.2–76.4).<br><br>• A total of 32,933 (39.0%) subjects were <70 years old, 25,145 (29.8%) were 70–74 years old, 15,758 (18.7%) were 75–79 years old, 7,715 (9.1%) were 80–84 years old, and 2,941 (3.5%) were 85+.<br><br>• There were 184 first episodes of VT-CAP or VT-IPD in the mITT population. A statistically significant vaccine-age interaction effect was observed (HR for interaction 1.057, 95% CI, 1.008–1.109, P = .023).<br><br>• The model-predicted that VE declined from 65% (95% CI, 38% to 81%) in 65-year-old subjects to 40% (95% CI, 17%; 56%) in 75-year-old subjects.<br><br>• Point estimates of the vaccine-age interaction effect were similar for VT-CAP and VT-IPD separately, for the PP analyses and after adjustment for covariates.	• Efficacy of PCV13 in preventing VT-CAP or VT-IPD was highest among subjects aged 65 at the time of randomization; VE declined with increasing age.
**Patterson et al. 2016 (3)**				
To quantify the persistence of VE of PCV13 in adults ≥65 years of age	**Post-hoc analysis of CAPiTA (whole cohort)**<br>see Bonten 2015<br><br>**Eligibility criteria**<br>see Bonten 2015	• The observed accumulation of first episodes for VT-CAP, VT-CAP (NB/NI-VT-CAP), and VT-IPD over the course of the study after vaccination was assessed.	• Cases of VT-CAP, NB/NI-VT-CAP, and VT-IPD were greater among PBO recipients compared with PCV13 recipients throughout the postvaccination observation period with a periodic rise in cases in the PBO group that was	• PCV13 was protective over the 5-year duration of the study, with no waning of efficacy observed.

Study objective(s)	Study details	Outcomes	Results	Conclusion by authors
		• Post hoc time-to-event analyses of primary (VT-CAP) and secondary (NB/NI VT-CAP or VT-IPD) endpoints were performed.<br><br>• VE behaviour over time was derived and effects of treatment, time, and time by treatment interactions were estimated.	consistent with varied exposure and ensuing disease over time.<br><br>• There was a significant difference in disease-free survival among PCV13 recipients compared with PBO recipients for VT-CAP (log-rank test P = 0.0005), NB/NI VT-CAP (P = 0.0051), and VT-IPD (P = 0.0004).<br><br>• VE ranged from 42.9% to 50.0% for VT-CAP, 36.2% to 48.5% for NB/NI-VT-CAP, and 66.7% to 75.0% for VT-IPD for each year over the 5 year study period.	
**van Werkhoven et al. 2016b (5)**				
To determine the accurateness of detecting CAP in the CAPiTA trial in which the NNT for prevention of VT pneumococcal CAP was 1,007 [95% CI: 613, 2,646]	**Substudy of CAPiTA (whole cohort)**<br>Data sources used:<br>1. Study database of identified episodes in 58/59 participating hospitals<br>2. GP records of participating subjects<br><br>GP records of participating subjects updated twice yearly with new information indicating hospital referral for (suspected) pneumonia were linked to the study database of episodes	• Two independent reviewers determined reasons for not identifying pneumonia episodes<br><br>• NNT adjusted for missed episodes was estimated.	• Of 2,183 hospital referrals with suspected pneumonia detected in GP records, 232 (11%) were admitted outside established screening routes and 102 (5%) were not suspected of pneumonia on admission.<br><br>• Of the remaining 1,849 episodes, 1,374 (63% of all episodes and 74% of identifiable episodes) were identified by RNs.<br><br>• After adjustment for missed episodes, the NNT reduced to 634 (95% CI: 386, 1,675).	• With the screening procedure, 63% of suspected pneumonia episodes were identified, and the estimated NNT reduced from 1,007 to 634.<br><br>• Several causes of missing episodes were identified, some of which appear preventable by adaptation of study procedures and training of study personnel.
**Huijts et al. 2017a (6)**				
To determine VE of PCV13 in prevention of a first episode VT-CAP in CAPiTA participants with DM, respiratory disease or heart disease at the time of vaccination.	**Post-hoc analysis of CAPiTA**<br>N= 40,427 older adults ≥65 years (subset of CAPiTA participants also included in Etio-CAP, an observational study conducted in parallel with CAPiTA)<br>○ N (PCV13): 20,196<br>○ N (PBO): 20,231<br><br>**Eligibility criteria -**<br>Presence of comorbidities at the time of vaccination based on documented comorbidity status in medical records.	• Presence of DM, heart disease, respiratory disease, liver disease, asplenia, and smoking at the time of immunization was verified on medical records in 139 subjects developing the primary endpoint of VT-CAP.<br><br>• Presence of DM and respiratory disease based on ICPC coding at GP was also determined in 40,427 subjects.	• In the 139 subjects developing VT-CAP, DM caused significant effect modification (p-value 0.002), yielding VE of 89.5% (95% CI, 65.5–96.8) and 24.7% (95% CI, -10.4 to 48.7) for those with and without DM, respectively.<br><br>• Comparable effect modification (p-value 0.020) was found in the 40,427 subjects with and without ICPC-based classification of DM with VE of 85.6% (95%CI, 36.7–96.7) and of 7.0% (95%CI, -58.5 to 45.5) respectively.	• Among immunocompetent elderly, VE of PCV13 was modified by DM with higher VE among subjects with DM.<br><br>• Significant effect modification was not observed for subjects with heart disease, respiratory disease,

Study objective(s)	Study details	Outcomes	Results	Conclusion by authors
		• The number of VT-CAP episodes, stratified by vaccine group and comorbidity status as defined in the VT-CAP dataset, was used to evaluate VE in people with and without each comorbidity.	• Effect modification through respiratory disease was not statistically significant, although the point estimate of VE was lower for those with respiratory disease in both analyses.<br><br>• There was no evidence of effect modification in subjects stratified by heart disease, smoking, and presence of any comorbidity.	smoking, or presence of any comorbidity.
**Huijts et al. 2017b (7)**				
To evaluate the efficacy of PCV13 vaccination in prevention of a first episode of IA-CAP or other virus-associated CAP (parainfluenza viruses, adenovirus, coronavirus, bocavirus, metapneumovirus, respiratory syncytial virus and rhinovirus) in CAPiTA participants	**Post-hoc analysis of CAPITA (whole cohort)** see Bonten (2015)<br><br>• After receiving PCV13 or PBO, study participants presenting with a clinical suspicion of pneumonia to any of the 59 participating sentinel centers (58 hospitals and one outpatient clinic) underwent a standardized clinical evaluation, including physical, radiological and microbiological evaluation, as well as an oropharyngeal swab and a urine sample for urinary antigen detection.	• CAP was defined by clinical and radiological criteria, and oropharyngeal swabs were collected from all individuals referred to a sentinel center with a clinical suspicion of pneumonia.<br><br>• Presence of influenza A and B, parainfluenza 1, 2, 3 and 4, human adeno-, boca-, corona-, metapneumo-, rhino- and respiratory syncytial viruses was determined by real-time PCR of oro-pharyngeal swab.	• Of 3209 episodes of suspected pneumonia, viral aetiology was tested in 2917 and proportions with influenza virus, human metapneumovirus and respiratory syncytial virus were 4.6%, 2.5% and 3.1%, respectively.<br><br>• There were 1653 oropharyngeal swabs for PCR testing available from 1814 episodes that fulfilled criteria for CAP, yielding 23 first episodes of IA-CAP in the PCV13 and 35 in the in PBO group - VE for IA-CAP of 34.4% (95% CI -11.1% to 61.2%; p 0.117).<br><br>• Annual influenza vaccination was received by 672 (87.2%) in the PCV13 group and 719 (87.7%) in the PBO group of the confirmed CAP cases	• PCV13 was not associated with a statistically significant reduction of influenza or virus-associated CAP.<br><br>• Overall incidence of non-influenza viral pneumonia was low.
**van Deursen et al. 2017 (8)**				
To determine long-term immunogenicity of PCV13 in pneumococcal vaccine–naive older adults as part of CAPiTA	**Substudy of CAPiTA**<br>N= 2011 CAPiTA study community-dwelling older adults aged ≥65 years participants<br>○ N (PCV13): 1006<br>○ N (PBO): 1005<br><br>A subset of 2011 subjects was enrolled in a single region between 15 September 2008 and 20 March 2009 at home visits.<br><br>**Eligibility criteria-**	• Immune responses to PCV13 before and at 1, 12, and 24 months after vaccination with 3 age-stratified study participant cohorts (65-69 years, 70-79 years, ≥80 years) were investigated.<br><br>• PCV13 serotype-specific OPA titres and IgG concentrations were determined.	• In all 3 age categories (65-69 years, 70-79 years, ≥80 years), a single dose of PCV13 elicited OPA titres and IgG concentrations for all 13 serotypes that were significantly higher than baseline and the corresponding responses in the PBO group at all time points.<br><br>• In the eldest subjects (≥80 years of age at vaccination), OPA titres and IgG concentrations remained above baseline, but were lower compared with younger	• A single dose of PCV13 elicits significant increases in OPA titres and IgG concentrations that persist 2 years postvaccination for all 13 serotypes, regardless of age and comorbidity<br>• Adults >=80 years at vaccination generally exhibited lower

Study objective(s)	Study details	Outcomes	Results	Conclusion by authors
	• Inclusion: No previous pneumococcal vaccination, self-reported immunocompetence<br>• Exclusion: Subjects with immunocompromising conditions<br><br>**Duration of Follow-up-**<br>24 months post vaccination<br><br>**Discontinuations over course of study, N (%)-**<br>○ PCV13: 115 (11.4%)<br>○ PBO: 122 (12.1%)<br><br>**Excluded from analyses, N (%)** – 56 (2.8%)<br>Most common reason for exclusion was absence of a blood sample within the required time period.		adults. There was no apparent difference in OPA titres and IgG concentrations between those with self-reported comorbidities and healthy older adults.	postvaccination anti-pneumococcal responses but levels remained above baseline.
**Webber et al. 2017 (9)**				
To assess the VE of PCV13 for the exploratory endpoints and<br>serotype distributions for pneumococcal CAP and IPD.	**Post-hoc analysis CAPiTA (whole cohort)**<br>see Bonten (2015)	• Exploratory CAP endpoints: first episode of confirmed non-VT (NVT) pneumococcal CAP; all confirmed episodes of NVT pneumococcal CAP, pneumococcal CAP, NB/NI VT pneumococcal CAP, and NB/NI pneumococcal CAP; and first and all episodes of culture-confirmed VT pneumococcal CAP, culture-confirmed pneumococcal CAP, culture confirmed NVT pneumococcal CAP, probable VT pneumococcal CAP, probable NVT pneumococcal CAP, and probable and possible pneumococcal CAP.<br><br>• Exploratory IPD endpoints included all episodes of VT-IPD and IPD, and first and all episodes of NVT-IPD.<br><br>• The PP and mITT populations were evaluated.	• Eight of 23 exploratory CAP and IPD endpoints were statistically significant (P<0.05) in both populations.<br><br>• In the PP population, these included VE of 28.9% (95% CI, 8.1, 45.1) for all episodes of confirmed pneumococcal CAP, 42.6% (95%CI: 11.7, 63.3) for all NB/NI episodes of VT pneumococcal CAP, 52.2% (95% CI: 17.1, 73.5) for all episodes of culture-confirmed pneumococcal CAP, and 52.6% (95% CI: 23.9, 71.2) for all episodes of IPD.<br><br>• Comparable VE estimates were observed in the mITT population.<br><br>• The most common VT serotypes were 1 (10 first episodes of confirmed pneumococcal CAP; 2 first episodes of IPD) and 7F (22; 7) among PCV13 and PBO recipients, respectively.	• The results of this analysis yielded statistically significant PCV13 VE for all episodes of confirmed pneumococcal CAP (including NB/NI and culture-confirmed episodes) and for all episodes of IPD.<br><br>• These findings are consistent with the primary CAPiTA trial efficacy analysis.
**Gessner et al. 2019 (10)**				
To assess the public health impact of PCV13 on	**Post-hoc analysis CAPiTA (whole cohort)**<br>See Bonten (2015)	Six clinical outcomes and three mortality outcomes were assessed.<br>○ Clinical CAP	• Results in order of clinical, adjudicated, Sp-CAP, and VT-Sp CAP; IPD and VT-IPD.	• A public health analysis of pneumonia and IPD outcomes found

Study objective(s)	Study details	Outcomes	Results	Conclusion by authors
clinically defined CAP, infection-related mortality; VPDIs and NNVs for all pneumonia and IPD outcomes; and PCV13-associated reductions in hospitalisation and ICU days.	Participants were classified by at-risk status based on self-reporting of any of the following chronic medical conditions: heart disease, lung disease, asthma, diabetes, liver disease, and smoking.	○ Adjudicated CAP (Clinical + radiologic infiltrate)<br>○ Sp-CAP (Adjudicated CAP + confirmed *S.pneumoniae* from sterile site or on UAD assay)<br>○ VT-CAP (Sp-CAP subset of patients with PCV13 VT)<br>○ IPD (*S.pneumoniae* from sterile site)<br>○ VT-IPD (Subset of IPD of patients with PCV13 VT)<br>○ Infection and infestation mortality (any infection related death)<br>○ All-cause mortality<br>○ Acute respiratory infection death<br><br>• For all outcomes, all randomised subjects were included, using mITT approach to determine VE, VPDI and NNV (based on a five-year duration of protection).	○ VEs (95% CI) for all hospital episodes were 8.1% (-0.6%, 16.1%), 6.7% (-4.1%, 16.3%), 22.2% (2.0%, 38.3%), 37.5% (14.3%, 54.5%), 49.3% (23.2%, 66.5%), and 75.8% (47.6%, 88.8%).<br><br>○ VPDIs per 100,000 PYOs were 72, 37, 25, 25, 20, and 15 with NNVs of 277, 535, 816, 798, 1016, and 1342.<br><br>• For clinical CAP, PCV13 was associated with a reduction of 909 (95% CI -115, 2013) hospital days per 100,000 PYOs translating to a reduction over 5 years of one hospital day for every 22 people vaccinated.<br><br>• When comparing at-risk persons to not at-risk persons, VEs were similar or lower, but because baseline incidences were higher the VPDIs were approximately 2–10 times higher and NNVs 50–90% lower.	substantial burden reduction following adult PCV13 immunization.<br><br>• VPDIs were higher among at-risk adults.
**Suaya et al., 2018 (11)**				
To assess VE of PCV13 against a first episode of VT-CAP in at-risk CAPITA participants	**Post-hoc analysis CAPiTA (whole cohort) See Bonten (2015)**<br><br>Participants were classified by at-risk status based on self-reporting of any of the following chronic medical conditions: heart disease, lung disease, asthma, diabetes, liver disease, and smoking.<br><br>**Included in post-hoc analysis, N (%) -**<br>○ PCV13: 42,019 (99.5%)<br>○ PBO: 42,045 (99.5%)<br><br>**At-risk subject, N (%)**<br>○ PCV13: 20,680 (49.2%)<br>○ PBO: 20,705 (49.2%)	Definition PP population and PP analysis see Bonten 2015	• PP analysis for a first episode of VT-CAP, PCV13 VE in at-risk participants was 40.3% (95% CI: 11.4%, 60.2%); in participants without known risk VE was 66.7% (95% CI: 11.8%, 89.3%).<br><br>• VE in overall study participants was 45.6% (95% CI: 21.9%, 62.5%).<br><br>• For all episodes of VT-CAP, PCV13 VE estimates were 36.5% (95% CI: 6.9%, 57.1%) in at-risk participants, 66.7% (95% CI: 11.8%, 89.3%) in those without known risk, and 42.4% (95% CI: 18.2%, 59.9%) in the overall study population.	• Results showed significant and persistent PCV13 VE against first and all episodes of VT-CAP in at-risk population.<br><br>• PCV13-induced protection did not wane over the course of the study.
**van Deursen et al. 2018 (12)**				
To determine the effects of PCV13 on nasopharyngeal	**Substudy of CAPiTA**<br>N = 2011 CAPiTA study community-dwelling older adults aged ≥65 years	• Subjects were vaccinated at home visit 1 (baseline) and followed up for 2 years with 3 home visits to collect	• Before randomisation and based on PCR, 339 of 1891 subjects had nasopharyngeal carriage with any	• PCV13 seemed to elicit a temporary reduction in VT nasopharyngeal

Study objective(s)	Study details	Outcomes	Results	Conclusion by authors
carriage of *S. pneumoniae*, *S. aureus*, *H. influenzae*, and *M. catarrhalis* in the first 24 months after vaccination in pneumococcal vaccine-naive CAPiTA participants	○ N (PCV13): 1006<br>○ N (PBO): 1005<br><br>A subset of 2011 subjects was enrolled in a single region between 15 September 2008 and 20 March 2009 at home visits.<br><br>**Eligibility criteria-**<br>○ Inclusion: No previous pneumococcal vaccination, self-reported immunocompetence<br>○ Exclusion: Subjects with immunocompromising conditions<br><br>**Duration of follow-up-**<br>24 months postvaccination<br><br>**Discontinuations, N (%)-**<br>PCV13: 47 (4.7%)<br>PBO: 59 (5.9%)<br><br>**Excluded from analyses, N (%) -** 112 (5.6%)<br>Most common reason for exclusion -absence of a valid postvaccination sample within the required time period (4.1% for PCV13 and 4.3% for PBO)	nasopharyngeal samples for carriage data at baseline and 6, 12, and 24 months after vaccination<br><br>• Serotyping of the 13 VT pneumococci was performed by PCR targeting capsular synthesis genes and Quellung reaction of isolates.	pneumococci (17.9%), and 114 of 1891 (6.0%) carried VT pneumococci. There was no difference between PCV13 and PBO groups.<br><br>• At 6 months after vaccination, VT pneumococcal carriage was significantly lower in PCV13 recipients than in the PBO group (RR, 0.53; 95% CI, 0.35–0.80; P = 0.04).<br><br>• There was no difference between the groups at 12 and 24 months after vaccination.<br><br>• Carriage of non-VT pneumococci, *S. aureus*, *H. influenzae*, and *M. catarrhalis* did not change between groups.	pneumococcal carriage.<br><br>• There were no clear changes in the prevalence of NVT pneumococci or other bacteria after PCV13 vaccination.
**Gessner et al. 2019b (13)**		•	•	•
To extend the original analysis of CAPiTA to include all CAP episodes regardless of radiological findings and include serotype results from all sources, including UAD, sterile sites, and non-sterile sites to determine serotype specific VE.	**Post-hoc analysis CAPiTA (whole cohort)**<br>*see Bonten 2015*<br><br>**Eligibility criteria:**<br>Any subject that met a clinical definition of CAP regardless of radiographic findings<br><br>**Clinical CAP –**<br>At least two of the following symptoms: cough, production of purulent sputum or a change in the character of sputum; temperature >38°C or <36.1°C; auscultatory findings consistent with pneumonia; leucocytosis (>10^*10^9 white blood cells/litre or >15% bands); C-reactive protein value >3 times the upper limit of normal; or hypoxemia	• VT-CAP was defined as any clinical CAP (regardless of radiographic findings) with a PCV13 serotype identified from culture of sterile or non-sterile (sputum and respiratory tract) sites, or from UAD.<br><br>• Both all episodes and first episodes were included.<br><br>• Only the five serotypes with at least 10 episodes in the control arm, based on the original analysis, were included for VE assessment.<br><br>• mITT population was analysed because the PP analysis, as defined in	• Of 272 clinical CAP visits with VT serotypes identified, 253 (93%) were identified by UAD including 210 (77%) by UAD alone.<br><br>• VE was determined for serotypes 1, 3, 6A, 7F, and 19A, with total first episodes of, respectively, 27, 36, 25, 38, and 48.<br><br>• VE (95% CI) for the five evaluated serotypes against first clinical CAP episodes were: serotype 1, 20.0% (95% CI:<br>-83.1% to 65.8%); serotype 3, 61.5% (95% CI: 17.6–83.4%); serotype 6A, 33.3% (95% CI: -58.6% to 73.2%); serotype 7F, 73.3%	• Statistically significant VE was observed for serotypes 3 and 7F for clinical CAP among elderly community dwelling adults.<br><br>• The VE point estimates and CIs for serotypes 1, 6A, and 19A were lower but consistent with the overall VT-CAP VE of 45.6% previously reported.<br><br>• These findings may be relevant in models to

Study objective(s)	Study details	Outcomes	Results	Conclusion by authors
	with a partial oxygen pressure >60 mmHg while breathing room air.<br><br>N= 84,496 older adults ≥65 years<br>○ N (PCV13): 42,240<br>○ N (PBO): 42,256<br><br>• Total visits for suspected pneumonia, N: 3209<br><br>• Visits meeting clinical CAP criteria, N: 3109<br>○ radiologic confirmation: 1935 (62%)<br>○ Not confirmed: 1174 (38%)	the original study protocol, required radiological confirmation of CAP episodes and the strict rule of identifying VT serotypes.	(95% CI: 40.5–89.4%); and serotype 19A, 45.2% (95% CI: -2.2% to 71.5%).	accurately account for the potential impact of adult PCV13 immunization alongside strong childhood PCV programs.
**Warren et al. 2020 (14)**				
To re-analyse published CAPiTA data on the efficacy of PCV13 against CAP caused by vaccine-targeted serotype using a hierarchical model that provides a potential framework for obtaining estimates of serotype-specific vaccine effects with reduced MSEs	**Post-hoc analysis CAPiTA (whole cohort)**<br>see Bonten 2015	• The CAPiTA data were from Bonten, et al. 2015 (1). The analyses focused on the data on number of cases of first episode non-bacteremic CAP caused by a serotype targeted by PCV13, according to the PP analysis.<br><br>• The responses from the vaccines and controls were considered as multinomial distributed vectors where each participant could end up in one of 14 different categories: with CAP caused by one of the 13 vaccine-targeted serotypes or as someone who did not develop CAP caused by a vaccine-targeted serotype.	• There were 61 cases of VT-pneumococcal pneumonia among the PBO recipients and 33 cases among the vaccinees.<br><br>• Without accounting for serotype, the estimated efficacy was 44.9% (95% (CrI): 20.6–66.8%). This is close to the efficacy estimate reported in the original study (45.0%, 95% CrI: 14.2–65.3%).<br><br>• **Serotype-specific efficacy, estimated separately -** The overall estimate of VE from this model was 46.1% (95% CrI: 21.2–67.2%).<br><br>• **Serotype-specific efficacy, estimated hierarchically -** The overall estimate of VE from this model was 46.0% (21.2–67.3%).	• Hierarchical modelling represents a useful approach for obtaining estimates of serotype-specific VE that are more stable for serotypes with small case counts and have reduced uncertainty than traditional unbiased estimators.<br><br>• This approach balances the need for serotype-specific estimates with the challenges of using sparse data and can be easily extended to alternative study designs.
**van Werkhoven et al. 2021 (15)**				
To evaluate the efficacy of PCV13 to prevent CAP and LRTI, LRTI-related antibiotic use, and total antibiotic use in primary care.	**Substudy of CAPiTA**<br>N= 40,426 older adults ≥65 years<br>○ N (PCV13): 20,195<br>○ N (PBO): 20,231<br><br>**Primary endpoint –**<br>CAP diagnosed and treated in primary care.	• PCV13 VE was determined using Poisson regression with robust standard errors, comparing CAP and non-CAP LRTI episodes, LRTI specific and total antibiotic prescriptions.	• A total of 1564 and 1659 CAP episodes occurred in the PCV13 and PBO group, respectively; VE 5.5% (95% CI -2.6% to 13.0%).<br><br>• Non-CAP LRTI episodes occurred 7535 and 7817 times in the PCV13 and PBO groups, respectively; VE 3.4% (95% CI -2.0% to 8.5%).	• PCV13 vaccination in the elderly is unlikely to cause a relevant reduction in the incidence of CAP, LRTI, LRTI-related antibiotic use or total antibiotic use in primary care.

Study objective(s)	Study details	Outcomes	Results	Conclusion by authors
	**Secondary endpoint(s) –**<br>Non-CAP LRTI treated in primary care, LRTI-related antibiotic use, and any antibiotic use.<br><br>**Additional outcome(s) –**<br>Clinically suspected CAP and LRTI in primary and secondary care combined.<br><br>The primary care data on incidence of CAP, LRTI and antibiotic prescriptions were collected from General Practice databases (EtioCAP study) (18).		• A total of 8835 and 9245 LRTI-related antibiotic courses were prescribed in the PCV13 and PBO arms, respectively; VE 4.2% (95% CI -1.0% to 9.1%).<br><br>• Antibiotic courses for any indication was prescribed 43,386 and 43,309 times, respectively; VE -0.4% (-4.9% to 3.9%).	

Abbreviations AIDS: Acquired immunodeficiency syndrome; CAP: Community acquired pneumonia; CI:Confidence interval; DM: Diabetes mellitus; GP: General practitioner; IgG: Immunoglobulin G; IA-CAP: Influenza associated community acquired pneumonia; ICPD: International Classification of Primary Care; ICU: Intensive Care Unit; IPD: Invasive pneumococcal disease; LRTI: Lower respiratory tract infection; mITT: Modified intention to treat; MSE: Mean square error; NB: Nonbacteremic; NI: Noninvasive; NVT: Non-vaccine type; NNT: Numbers needed to treat; NNV: Numbers needed to vaccinate; OPA: Opsonophagocytic activity; PYO: Patient years; PP: Per protocol; PBO: Placebo; PCV: Pneumococcal conjugate vaccine; PCR: Polymerase chain reaction; Sp: S.pneumoniae; SAE: Serious adverse event; SS: Serotype-specific; CAPiTA: The Community-Acquired Pneumonia immunization Trial; UAD: Urinary antigen detection; VE: Vaccine efficacy; VPDI: Vaccine-preventable disease incidence; VT: Vaccine-type

References

1. Bonten MJ, Huijts SM, Bolkenbaas M, Webber C, Patterson S, Gault S, et al. Polysaccharide conjugate vaccine against pneumococcal pneumonia in adults. N Engl J Med. 2015;372(12):1114-25.
2. van Werkhoven CH, Huijts SM, Bolkenbaas M, Grobbee DE, Bonten MJ. The Impact of Age on the Efficacy of 13-valent Pneumococcal Conjugate Vaccine in Elderly. Clin Infect Dis. 2015;61(12):1835-8.
3. Patterson S; Webber C; Patton M; Drews W; Huijts SM.; Bolkenbaas M et al. A post hoc assessment of duration of protection in CAPiTA (Community Acquired Pneumonia immunization Trial in Adults. Trials in Vaccinology 2016;5:92-6.
4. van Werkhoven CH, Hollingsworth RC, Huijts SM, Bolkenbaas M, Webber C, Patterson S, Sanders EA, Bonten MJ. Pneumococcal conjugate vaccine herd effects on non-invasive pneumococcal pneumonia in elderly. Vaccine. 2016;34(28):3275-82.
5. van Werkhoven CH, Huijts SM, Paling FP, Bonten MJ. The scrutiny of identifying community-acquired pneumonia episodes quantified bias in absolute effect estimation in a population-based pneumococcal vaccination trial. J Clin Epidemiol. 2016;69:185-92.
6. Huijts SM, van Werkhoven CH, Bolkenbaas M, Grobbee DE, Bonten MJM. Post-hoc analysis of a randomized controlled trial: Diabetes mellitus modifies the efficacy of the 13-valent pneumococcal conjugate vaccine in elderly. Vaccine. 2017;35(34):4444-4449.
7. Huijts SM, Coenjaerts FEJ, Bolkenbaas M, et al. The impact of 13-valent pneumococcal conjugate vaccination on virus-associated community-acquired pneumonia in elderly: Exploratory analysis of the CAPiTA trial. Clin Microbiol Infect. 2018;24(7):764-770.
8. van Deursen AMM, van Houten MA, Webber C, Patton M, Scott DA, Patterson S, Sidhu M, Drews W, Gruber WC, Emini EA, Grobbee DE, Bonten MJM, Sanders EAM. Immunogenicity of the 13-Valent Pneumococcal Conjugate Vaccine in Older Adults With and Without Comorbidities in the Community-Acquired Pneumonia Immunization Trial in Adults (CAPiTA). Clin Infect Dis. 2017;65(5):787-795.
9. Webber C, Patton M, Patterson S, Schmoele-Thoma B, Huijts SM, Bonten MJ; CAPiTA Study Group. Exploratory efficacy endpoints in the Community-Acquired Pneumonia Immunization Trial in Adults (CAPiTA). Vaccine. 2017;35(9):1266-72.
10. Gessner BD, Jiang Q, Van Werkhoven CH, Sings HL, Webber C, Scott D, Neuzil KM, O'Brien KL, Wunderink RG, Grobbee DE, Bonten MJM, Jodar L. A public health evaluation of 13-valent pneumococcal conjugate vaccine impact on adult disease outcomes from a randomized clinical trial in the Netherlands. Vaccine. 2019;37(38):5777-87.
11. Suaya JA, Jiang Q, Scott DA, Gruber WC, Webber C, Schmoele-Thoma B, Hall-Murray CK, Jodar L, Isturiz RE. Post hoc analysis of the efficacy of the 13-valent pneumococcal conjugate vaccine against vaccine-type community-acquired pneumonia in at-risk older adults. Vaccine. 2018;36(11):1477-83.
12. van Deursen AMM, van Houten MA, Webber C, Patton M, Scott D, Patterson S, Jiang Q, Gruber WC, Schmoele-Thoma B, Grobbee DE, Bonten MJM, Sanders EAM. The Impact of the 13-Valent Pneumococcal Conjugate Vaccine on Pneumococcal Carriage in the Community Acquired Pneumonia Immunization Trial in Adults (CAPiTA) Study. Clin Infect Dis. 2018;67(1):42-9.
13. Gessner BD, Jiang Q, Van Werkhoven CH, Sings HL, Webber C, Scott D, Gruber WC, Grobbee DE, Bonten MJM, Jodar L. A post-hoc analysis of serotype-specific vaccine efficacy of 13-valent

pneumococcal conjugate vaccine against clinical community acquired pneumonia from a randomized clinical trial in the Netherlands. Vaccine. 2019;37(30):4147-54.
14. Warren JL, Weinberger DM. Estimating Serotype-specific Efficacy of Pneumococcal Conjugate Vaccines Using Hierarchical Models. Epidemiology. 2020 Mar;31(2):259-62.
15. van Werkhoven CH, Bolkenbaas M, Huijts SM, Verheij TJM, Bonten MJM. Effects of 13-valent pneumococcal conjugate vaccination of adults on lower respiratory tract infections and antibiotic use in primary care: secondary analysis of a double-blind randomized PBO-controlled study. Clin Microbiol Infect. 2021 Jul;27(7):995-999. doi: 10.1016/j.cmi.2020.09.011. Epub 2020 Sep 22. PMID: 32971253.
